# Rescue of Primary Incomplete Microkeratome Flap with Secondary Femtosecond Laser Flap in LASIK

**DOI:** 10.1155/2014/289354

**Published:** 2014-11-23

**Authors:** E. A. Razgulyaeva

**Affiliations:** Tyumen Regional Ophthalmological Treatment Center, Tyumen 625000–625010, Russia

## Abstract

For laser-assisted in situ keratomileusis (LASIK) retreatments with a previous unsuccessful mechanical microkeratome-assisted surgery, some surgical protocols have been described as feasible, such as relifting of the flap or the creation of a new flap and even the change to a surface ablation procedure (photorefractive keratectomy (PRK)). This case shows the use of femtosecond technology for the creation of a secondary flap to perform LASIK in a cornea with a primary incomplete flap obtained with a mechanical microkeratome. As we were unable to characterize the interface of the first partial lamellar cut, a thick flap was planned and created using a femtosecond laser platform. As the primary cut was very thick in the nasal quadrant, a piece of loose corneal tissue appeared during flap lifting which was fitted in its position and not removed. Despite this condition and considering the regularity of the new femtosecond laser cut, the treatment was uneventful. This case report shows the relevance of a detailed corneal analysis with an advanced imaging technique before performing a secondary flap in a cornea with a primary incomplete flap. The femtosecond laser technology seems to be an excellent tool to manage such cases successfully.

## 1. Introduction

Femtosecond laser technology has supposed a great advance for flap creation in LASIK surgery and has demonstrated advantages over conventional mechanical microkeratomes [1, 2]. The use of mechanical microkeratomes has been shown to be associated with higher rates of epithelial defects [3], displacement of the flap [4], and epithelial ingrowth [5]. Nevertheless, LASIK procedures performed with mechanical microkeratomes have been widely used for years and thus some patients may need a secondary laser treatment as a consequence of an over- or undercorrection, or regression [6]. In such cases refractive surgeons may perform a photorefractive keratectomy (PRK) retreatment, and also a LASIK retreatment with relifting of the original flap or the creation of a new flap in a deeper plane [7]. If flap relifting is not possible, the use of femtosecond technology for the creation of the secondary flap is recommendable due to its high level of predictability with regards to flap depth and geometry. However, a comprehensive preretreatment analysis of the cornea by means of an imaging technique is necessary to avoid a flap cut interfering with the original cut, especially if this cut is irregular or incomplete due to intraoperative complications. This case report shows the relevance of this detailed corneal analysis in a case of a secondary femtolaser flap in a cornea with a primary incomplete microkeratome flap.

## 2. Case Report

A 28-year-old female presented herself at our clinic for refractive laser correction. Her preoperative refraction was −3.75 (sph), −0.75 (cyl) at 176° in the right eye (RE) and −3.75 (sph), −0.5 (cyl) at 5° in the left eye (LE). The corrected distance visual acuity (CDVA) was 20/20 and 20/20 in RE and LE, respectively. After a comprehensive ophthalmological examination, LASIK was considered the most adequate surgical option for the treatment of her refractive error. The flap was created using a mechanical microkeratome (Moria M2 Single Use, Moria SA, Antony, France) with a disposable and calibrated Med-Logics minus-20 blade (Med-Logics Inc., CA, USA). A flap head of 100 *μ*m of thickness was used. Surgery was uneventful in RE but a partial cut with the microkeratome was performed in LE due to a loss of suction, and consequently the laser treatment was aborted.

Three months after this episode, when the refraction and the topography were stable, an analysis of the LE cornea was performed with the optical coherence tomography (OCT) system Cirrus HD-OCT (Carl Zeiss Meditec, Germany) in order to determine the position and trajectory of the irregular cut, but the interface of the incomplete primary flap was not visible, even with high magnification. Considering that the primary intended flap had a theoretical central thickness of 100 *μ*m, a new cut using the IntraLase femtosecond laser FS-60 (Abbott Medical Optics, Inc.) was performed using the following settings: diameter of 9 mm and flap thickness of 140 *μ*m. This planning was aimed at avoiding an interference with the primary incomplete cut. Although the planning was 140 *μ*m, according to the OCT image, the flap was approximately of 100–110 *μ*m in the center. There was no subepithelial opacity that may have predisposed to an incomplete lamellar pass due to the vertical femtosecond gas breakthrough.

**Figure 1:**
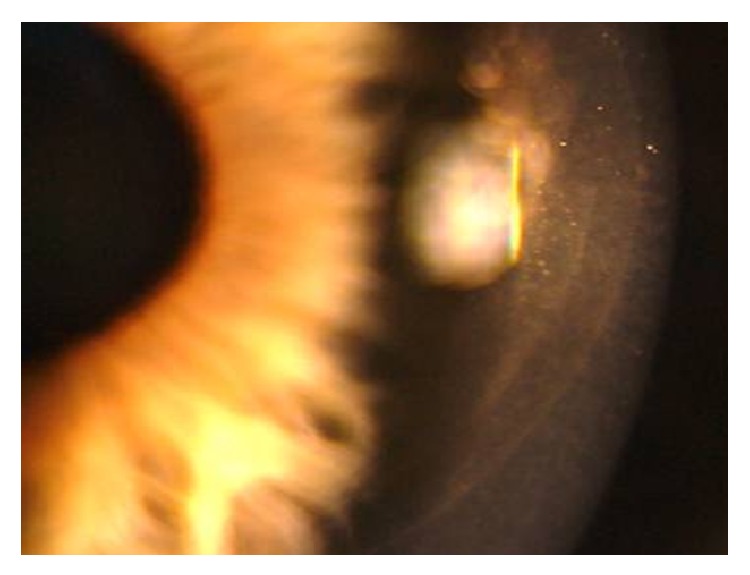
Slit-lamp visualization of both flap edges.

**Figure 2:**
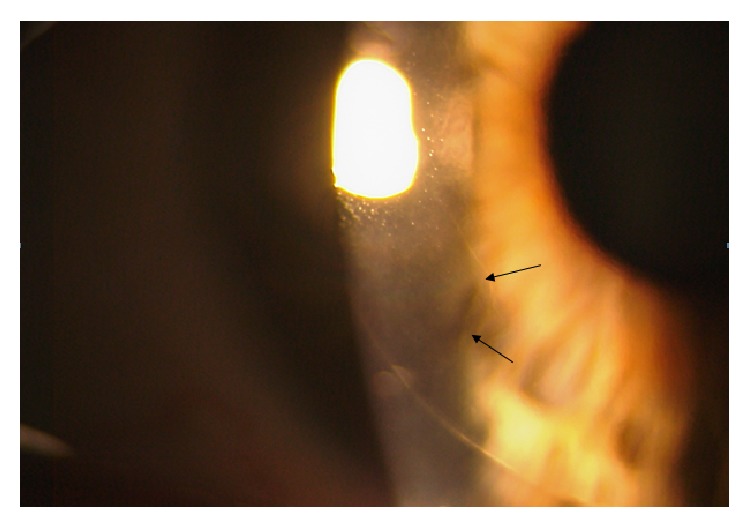
Delimitation (black arrows) of the loose corneal tissue that was present in the nasal quadrant and evidenced after lifting the flap.

**Figure 3:**
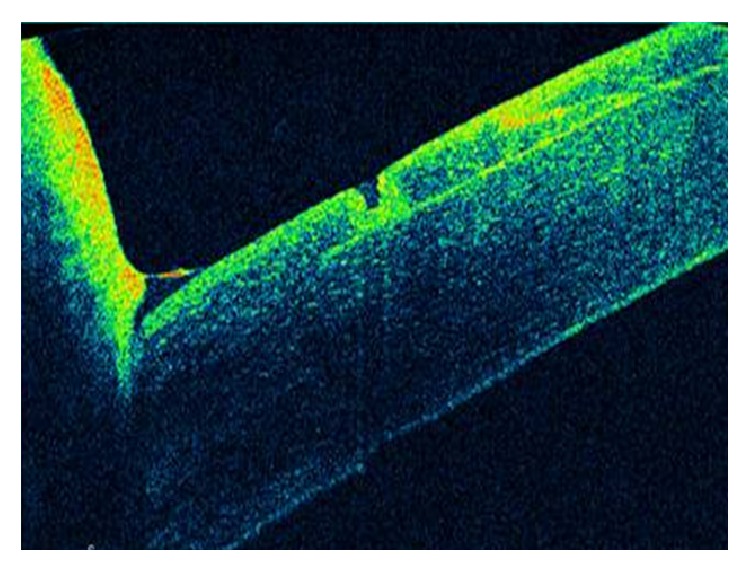
In the lower quadrant, the cut done primarily with the microkeratome opened up from inside after colliding with the new femtosecond flap, leading to an epithelial defect that was visualized with the OCT system.

**Figure 4:**
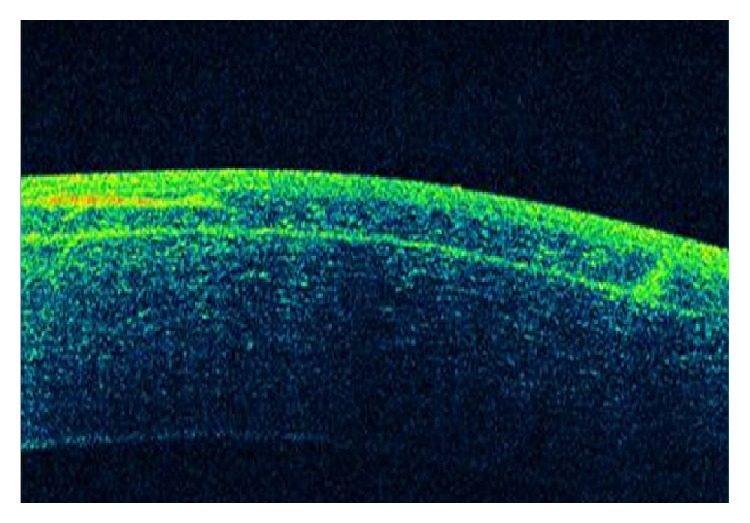
OCT image of both flaps in the temporal quadrant showing the abrupt end of the primary cut done with the mechanical microkeratome.

At the immediate postoperative visit, the slit-lamp examination allowed the visualization of both flap edges (Figure 1). Intraoperatively, a piece of loose corneal tissue was observed in the nasal quadrant after lifting the flap which was thought to be the result of the presence of a primary cut of more than 140 *μ*m at this position. This piece of corneal tissue was fitted in its position and not removed. Its extension could be also delimited at the slit lamp (Figure 2). In the lower quadrant, the primary microkeratome cut opened up from inside after colliding with the new femtosecond flap, leading to an epithelial defect (Figure 3). In the temporal quadrant, both flaps were clearly positioned at different levels and the abrupt end of the primary mechanical cut was visible in the OCT analysis (Figure 4). Despite this condition and considering the regularity of the new femtosecond cut, laser treatment was uneventfully applied. The day after surgery, the patient achieved an uncorrected distance visual acuity (UDVA) of 0.9 (decimal notation).

## 3. Discussion

As shown in this case report, femtosecond laser-assisted LASIK is a safe option to manage unsuccessful previous LASIK procedures with mechanical microkeratomes. If the primary flap has been uneventfully done and relifting with manual dissection is not possible because of its strong adhesion, the creation of a new vertical side cut with the use of the femtosecond laser creates a net interface that diminishes the mechanical trauma to the epithelium (Güell et al. [8]) and prevents epithelial ingrowth due to the vertical side cut configuration of the femtosecond flap compared to the sloping edge of the mechanical-based flap [9]. Vaddavalli et al. found that femtosecond retreatment using a side cut-only algorithm is effective in the treatment of residual errors after mechanical microkeratome-based LASIK, reducing the rates of epithelial ingrowth [10]. The technique from Güell et al. [8] was shown to be also useful if the primary cut is irregular or incomplete, as in our case.

The preoperative assessment of the primary flap through an imaging technique, such as OCT, is essential, especially in the peripheral area, where both primary and secondary flaps are expected to interfere due to the wide variability of thickness of a flap obtained with a mechanical microkeratome [11–13]. Anterior segment OCT (AS-OCT) is an imaging technology that would provide enough information about the characteristics of the primary flap in cases like that presented here [14–16], but in some cases the difficulty in detecting the interface can be significant. This difficulty is especially relevant when a posterior segment OCT is used for the analysis of the cornea because of the unavailability of an AS-OCT at the clinical setting, as happened in our case. This inability of characterizing the interface prior to the second cut can lead to complications such as the presence of some loose tissue, scars, or loss of a sliver of tissue. Pietilä et al. used the FEMTO LDV femtosecond laser (Ziemer Ophthalmic Systems, Port, Switzerland) to perform a new thin flap (90 *μ*m) in 81 eyes previously operated with a mechanical microkeratome. They found an improved predictability in flap thickness with their cutting and a low rate of complications such as adhesions related to scars of the primary flap, with no further prevention of the excimer laser ablation treatment. However, these authors did not recommend the creation of a new femtosecond flap in cases of primary free cap or visible scars in the old flap [17]. Garcia-Gonzalez and Teus created a new femtosecond mini-flap, smaller in diameter compared to the previous one, in 10 eyes of 7 patients to avoid the adhesion of the primary flap edges, and without interferences with the peripheral interface, preventing the risk of dislocation of the primary flap [18]. In our case, the lack of an accurate OCT image delimiting the previous partial flap led us to program a new thick femtosecond flap, assuming a higher risk of interference between the primary and secondary flap in the peripheral cornea and consequently to the generation of loose tissue at this area. Fortunately, this complication was easily managed with no further consequences. In conclusion, femtosecond laser-assisted LASIK retreatment in eyes with previous unsuccessful surgery with the creation of an incomplete or irregular flap using a mechanical microkeratome seems to be a safe and highly predictable option. It should be considered that an incomplete microkeratome flap results in an irregular topographic pattern [19, 20] that should be treated by means of topography or wavefront-guided procedures in order to minimize the higher order aberrations and corneal irregularity induced with the incomplete cut [21–24]. In such cases, it is highly recommended to perform a complete and detailed analysis of the primary flap through AS-OCT imaging technology [25, 26] prior to the retreatment in order to define the most appropriate planning for the lamellar cut. Likewise, AS-OCT is able to evaluate epithelial remodeling which can be considered another important biological metric to take into account when evaluating this or any similar complicated refractive surgery cases [25].
